# Editorial: Highlights in psychology for clinical settings: the ascent of digital psychotherapy

**DOI:** 10.3389/fpsyg.2023.1275034

**Published:** 2023-08-31

**Authors:** Moshe Bensimon, Meyran Boniel-Nissim, Vera Békés, Eamonn Patrick Arble, Keren Gueta

**Affiliations:** ^1^Department of Criminology, Bar-Ilan University, Ramat Gan, Israel; ^2^Department of Educational Counseling, Max Stern Academic College of Emek Yezreel, Emek Yezreel, Israel; ^3^Ferkauf Graduate School of Psychology, Yeshiva University, New York, NY, United States; ^4^Psychology Department, Eastern Michigan University, Ypsilanti, MI, United States

**Keywords:** digital psychotherapy, telepsychotherapy, remote psychotherapy, online therapy, videoconferencing psychotherapy, telemental health, COVID-19 pandemic

Traditional face-to-face treatment sessions conducted within the boundaries of a therapist's office have long represented the primary practice of mental health. However, since the COVID-19 pandemic outbreak, digital evolution has introduced intensive changes in psychotherapy. Digital psychotherapy (DP), also often known as telepsychotherapy, remote psychotherapy, online therapy, videoconferencing psychotherapy or telemental health, uses technological advances to provide mental health services outside the traditional in-person synchronous therapeutic context. This Research Topic, *Highlights in psychology for clinical settings: the ascent of digital psychotherapy*, examines aspects of DP and emphasizes the need to further develop this transformative approach to mental health care.

The Research Topic consists of 13 original articles focusing on various aspects of DP, including challenges and opportunities, the efficacy of DP from patients' and psychotherapists' perspectives, and mechanisms of change. Challenges and opportunities are discussed in Fernández-Álvarez and Fernández-Álvarez's perspective article about the therapeutic alliance and adapting interventions to fit the patient's preferences. Additionally, this paper outlined how the field of psychotherapy could benefit from the unprecedented situation presented by the COVID-19 pandemic. Gueta et al.'s qualitative pilot study examined the cultural accommodation of an existing Internet intervention for substance use and related disorders in Israel. Thematic analysis of interviews with patients and therapists and a literature review yielded a comprehensive cultural accommodation framework.

Several articles explore the efficacy of online therapy modalities and techniques. Sperandeo et al.'s preliminary study examined the digital empathy gap in DP between Italian psychotherapist and patient dyads, showing that unlike psychotherapists, patients perceived their therapists as significantly more empathic and supportive in the remote setting compared to perceived empathy in in-person settings. From the psychotherapists' perspective, Stefan et al.'s longitudinal mixed-methods study showed that remote psychotherapy in Austria could be a credible and trustworthy alternative to in-person treatment, which most psychotherapists could adopt and implement regardless of theoretical orientation. Reatto et al. showed that Italian patients with insecure attachment styles had greater difficulties adapting to DP, thus confirming that insecure attachment is a vulnerability factor for psychopathological problems and for a well-functioning therapeutic collaboration. Finally, Békés et al. presented a validation of the Teletherapy Intervention Scale for clinicians and researchers; the scale was found to be positively related to the working alliance, the real relationship, and the therapeutic presence in teletherapy sessions, as well as to positive attitudes toward teletherapy and intention to use teletherapy in the future.

From the patient's perspective, the experience of DP was complex and nuanced. Werbart et al.'s qualitative study in Sweden showed that some aspects of switching from face-to-face to DP were perceived as harming the quality of treatment, whereas other patients may experience more freedom in DP compared to an in-person setting. Their findings indicated the importance of considering patient characteristics when transitioning to DP formats. Harris et al.'s brief research report found that the average outcome trajectory for patients in the USA who received DP was statistically like those in an earlier cohort who received in-person services before the COVID-19 pandemic onset. Wesołowski et al.'s thematic analysis with Polish potential therapy patients indicated that DP frustrated the need for psychological contact, contributed to negative emotions, but sometimes was perceived as better than in-person therapy. DP served as a solution during the pandemic by providing a sense of continuity during a lockdown and enabling adapting to exceptional circumstances; however, some participants expressed concerns about the effectiveness of DP and its credibility. von Below et al.'s thematic analysis of interviews with Swedish patients' experiences of transition to DP at the start of the pandemic and then later back to the office indicated that the patients experienced the process in DP as impeded. Patients reported that emotional expression was hampered by the loss of non-verbal communication, the emotional relationship was altered, and at the same time, DP allowed the patients to incorporate therapy more into their everyday lives. In a brief research report, Drüge et al. used a mixed methods study to investigate whether innovative moments (IMs) occur in Swiss patients' telephone-based cognitive-behavioral therapy (t-CBT). They examined the association between IMs and symptom improvement, reconceptualization, and symptom improvement and found that IMs also occur in t-CBT and can be reliably rated by external observers.

Some papers in the present Research Topic also discussed mechanisms of change in therapy. Several of these mechanisms are a product of the translation of in-person techniques to a digital format, and some are unique to the digital arena. In this context, Domhardt et al., in their perspective article, highlighted the increased opportunity to conduct more precise psychotherapy process research to understand change mechanisms that were only feasible with digital tools and outlined essential future directions for this novel branch of research. Furthermore, they indicated several challenges and pitfalls to be solved to advance DP research. Another reviewed digital tool was the incorporation of virtual reality (VR) technology into DP. In their brief research report, Horigome et al. conducted a feasibility study in Japan using VR technology in the framework of exposure therapy with four patients with social anxiety disorder. This feasibility study hints at the possibility of incorporating new technologies into digital clinical work.

To conclude, DP represents a revolutionary paradigm shift in mental health care, emerging as a significant tool during times of crisis (such as natural disasters or pandemics) and, increasingly, as a standard treatment model. However, several areas of research within DP require more empirical attention. First, more research is needed regarding the mechanisms of change in DP, such as therapeutic alliance, power balance, and empathic accuracy, which may play different roles in DP compared to in-person therapy. Second, a better understanding of the comparative efficacy between traditional and digitalized therapy techniques is needed. Finally, more research is necessary regarding tailoring procedures to patients' needs, such as personal characteristics and cultural background. Such data can inform personalized treatment plans, optimize the therapeutic process and enhance the overall efficacy of mental health interventions through DP.

## Author contributions

MB: Conceptualization, Project administration, Writing—original draft, Writing—review and editing. MB-N: Writing—review and editing. VB: Writing—review and editing. EA: Writing—review and editing. KG: Conceptualization, Writing—original draft, Writing—review and editing.

